# Opportunistic screening for osteoporosis by abdominal CT in a British population

**DOI:** 10.1186/s13244-023-01400-1

**Published:** 2023-04-01

**Authors:** Sonam Vadera, Timothy Osborne, Vikas Shah, James A. Stephenson

**Affiliations:** Gastrointestinal Imaging Group, Department of Radiology, University Hospitals of Leicester, Leicester General Hospital, Leicester, UK

**Keywords:** Osteoporosis, Dual-energy X-ray absorptiometry, Computed tomography, Screening

## Abstract

**Background:**

It has previously been shown that CT scans performed for other indications can be used to identify patients with osteoporosis. This has not yet been tested in a British population. We sought to evaluate the use of vertebral CT attenuation measures for predicting osteoporosis in a British cohort, using dual-energy X-ray absorptiometry (DEXA) as a reference standard.

**Methods:**

Patients who underwent abdominal CT in 2018 and concomitantly underwent DEXA within a six-month interval were retrospectively included. CT attenuation values in Hounsfield units (HU) were measured by placement of a region-of-interest at the central portion of the L1 vertebral body and then compared to their corresponding DEXA score. Receiver operating characteristic (ROC) curves were generated to evaluate the performance of a logistic regression model and to determine sensitivity and specificity thresholds.

**Results:**

536 patients (394 females, mean age 65.8) were included, of which 174 had DEXA-defined osteoporosis. L1 attenuation measures were significantly different (*p* < 0.01) between the three DEXA-defined groups of osteoporosis (118 HU), osteopenia (143 HU) and normal bone density (178 HU). The area under the ROC curve was 0.74 (95% CI 0.69–0.78). A threshold of 169 HU was 90% sensitive, and a threshold of 104 HU was 90% specific for diagnosing osteoporosis.

**Conclusions:**

Routine abdominal CT can be used to opportunistically screen for osteoporosis without additional cost or radiation exposure. The thresholds identified in this study are comparable with previous studies in other populations. We recommend radiologists engage with primary care and rheumatology providers to determine appropriate cut-off values for further investigation.

## Key points

1. Vertebral body attenuation measurements can be used to screen for osteoporosis.
2. This is the first study to confirm the applicability to a British population.
3. This opportunity to detect osteoporosis earlier is without additional radiation exposure.
4. A threshold of 169 HU was 90% sensitive for diagnosing osteoporosis.
5. Thresholds could be refined based on local population risk profiles.

## Background

Osteoporosis is defined as a ‘systemic skeletal disease characterised by low bone mass and microarchitectural deterioration of bone tissue, with a consequent increase in bone fragility and susceptibility to fracture’, and is estimated to affect more than 200 million people worldwide [1, 2]. The condition is a major cause of morbidity and mortality. It is also associated with a significant economic burden; costs of caring for patients with osteoporosis-related fractures have been shown to parallel or exceed that of other serious diseases, including myocardial infarctions and cerebrovascular accidents [3–5]. Osteoporosis is usually an asymptomatic condition, only manifesting clinically after the occurrence of a fracture. Early diagnosis can enable initiation of treatment to target the disease during this asymptomatic phase and thus reduce fracture risk. However, despite being a prevalent and treatable condition with diagnostic modalities available for screening, osteoporosis unfortunately remains under-treated and under-diagnosed [6–8]. Whilst substantial advances in assessment and treatment of osteoporosis have emerged in recent decades, rapid ageing of the global population combined with overstrained health services has resulted in a persistent ‘treatment gap’ in patients with osteoporosis, necessitating novel and opportunistic methods to identify patients at high risk of fragility fractures [9, 10].

The current gold standard tool recognised for diagnosis and monitoring of osteoporosis is dual-energy X-ray absorptiometry (DEXA) of the lumbar spine and hips [11]. However, this is often underused and also presents its own limitations [11, 12]. DEXA scans frequently produce false negative results in patients with osteoporosis-related vertebral compression fractures [13–15], and measurements may also be skewed by various factors including sclerotic bony lesions, vascular calcifications, and increased body fat [16].

In recent years, it has been increasingly recognised that routine CT scans performed for other purposes could be used to identify patients with osteoporosis [14, 15, 17–22]. Abdominal CT scans are widely performed for a wide variety of indications and contain rich amounts of data for evaluation of the lumbar spine. However, with no clear guidance available, these valuable data are frequently overlooked, leading to missed opportunities for early diagnosis of osteoporosis.

Previous work has suggested that measures of trabecular bone attenuation using a simple region-of-interest (ROI) measure can enable rapid and straightforward assessment of bone mineral density without any additional radiation exposure to the patient, thus enabling earlier detection of osteoporosis [21]. However, this has not yet been tested in a British population. The main purpose of this study was to evaluate the utility of routine abdominal CT performed for other indications in opportunistically screening for osteoporosis in a British population, using DEXA as a reference standard.

## Methods

Patients over the age of 18 who underwent an abdominal CT scan in 2018 and who concomitantly underwent a DEXA scan within six months (before or after the CT) were retrospectively included in the study. Given the retrospective nature of this study, ethical approval was not required. However, approval from the local audit and quality improvement committee was obtained.

### DEXA

DEXA scans were performed on the lumbar spine, proximal femora and radius following standard protocol using GE lunar iDXA scanners. For inclusion, at least one valid T-score from the lumbar spine, proximal femora or radius was required. In accordance with standard recommendations [22], patients were classified into three groups according to their lowest reported T-score at any measured location: normal (T-score ≥ −1.0), osteopenia (−2.5 < T-score < −1.0) and osteoporosis (T-score ≤ −2.5).

### CT

Abdominal CT scans performed for other indications were acquired using standard techniques on one of ten multi-detector CT scanners at three hospital sites. All parameters from various protocols were accepted to mirror real-life scanning.

**Fig. 1:**
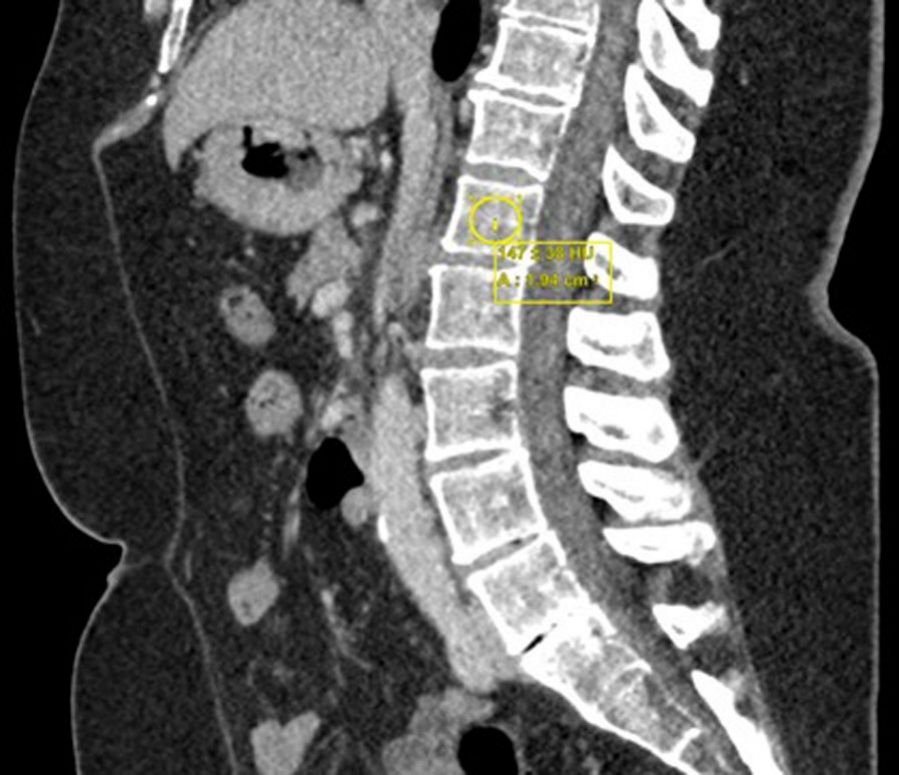
Example of ROI placement over the L1 vertebra

CT images were retrospectively analysed on a standard radiology picture archiving and communication system. Attenuation values were measured in Hounsfield units (HU) from the L1 vertebra on the sagittal reconstruction only, with lower HU values representing lower bone mineral density. The L1 vertebra was used as it is included on most thoracic and abdominal CT scans and is easily identifiable. A click-and-drag oval region of interest (ROI) was placed in the central portion of the vertebral body over an area of trabecular bone (Fig. 1). Cortical bone, fractures and areas of focal lesion or heterogeneity (such as haemangiomas, bone islands*,* artefacts, and the posterior venous plexus) were avoided in order to prevent distortion of the attenuation values. Three ROI measurements were taken to obtain a mean attenuation value. Where a reliable L1 attenuation measurement was not feasible (e.g. due to a compression fracture or focal lesion), the nearest unfractured, visible vertebra was utilised instead.

### Statistical analysis

One-way analysis of variance (ANOVA) tests were performed to compare mean CT attenuation values between the three DEXA-defined categories. Correlation between CT attenuation values and DEXA T-scores was assessed using the Pearson correlation coefficient.

CT performance was assessed using receiver operating characteristic (ROC) curve analysis. We calculated sensitivity, specificity, negative predictive value (NPV) and positive predictive value (PPV) and area under the receiver operator characteristic curve (AUC) for two different pre-defined thresholds that have previously been proposed [14]. We also determined optimal HU thresholds in this patient cohort from a univariate logistic regression model.

## Results

The study comprised of 536 patients (394 [73.5%] females) with a mean (SD) age of 65.8 (13.0). Of these, 174 (32.5%) had osteoporosis, 220 (41.0%) had osteopenia, and 142 (26.5%) had a normal bone mineral density (BMD) as defined by the DEXA reference standard. The mean interval between CT and DEXA scan was 89.5 days (Table 1).

**Table 1:** Characteristics of patients included with and without DEXA-defined osteoporosis

Variable	Osteoporosis	Non-osteoporosis
Number of patients	174	362
Age (Mean years (SD))	69 (12)	64 (13)
Sex (N (%) females)	134 (77%)	260 (72%)
Lowest DEXA T-score (Mean (SD))	−3.27 (0.71)	−1.09 (1.15)
Days between CT and DEXA (Mean (SD))	87.6 (47.5)	90.4 (51.7)
CT attenuation in HU (Mean (SD))	118.5 (40.2)	156.6 (51.3)

*CT* computed tomography, *DEXA* dual-energy X-ray absorptiometry, *HU* Hounsfield units, *SD* standard deviation

**Fig. 2:**
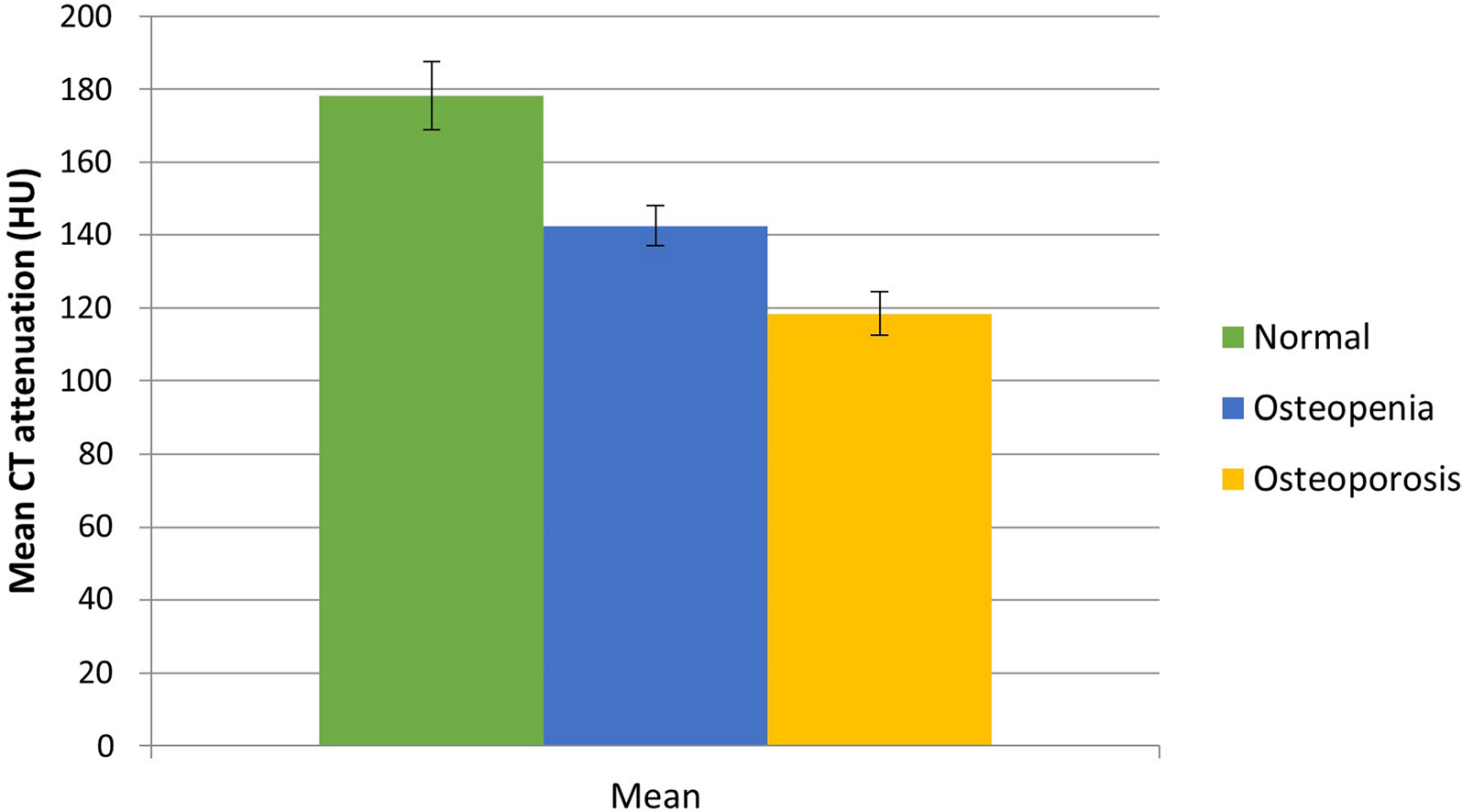
Mean CT attenuation values (measured in HU), stratified according to DEXA reference standard as osteoporosis, osteopenia or normal BMD. Black vertical lines represent 95% CIs. The differences in mean CT attenuation are significantly different between each subgroup (*p* < 0.01)

The mean HU values were 118 HU (95% CI 112–124 HU) in the osteoporosis subgroup; 143 HU (95% CI 137–148 HU) in the osteopenia subgroup; and 178 HU (95% CI 169–188 HU) in the normal BMD subgroup. The difference in mean CT attenuation between each subgroup was statistically significant (*p* < 0.01) (Fig. 2).

**Fig. 3:**
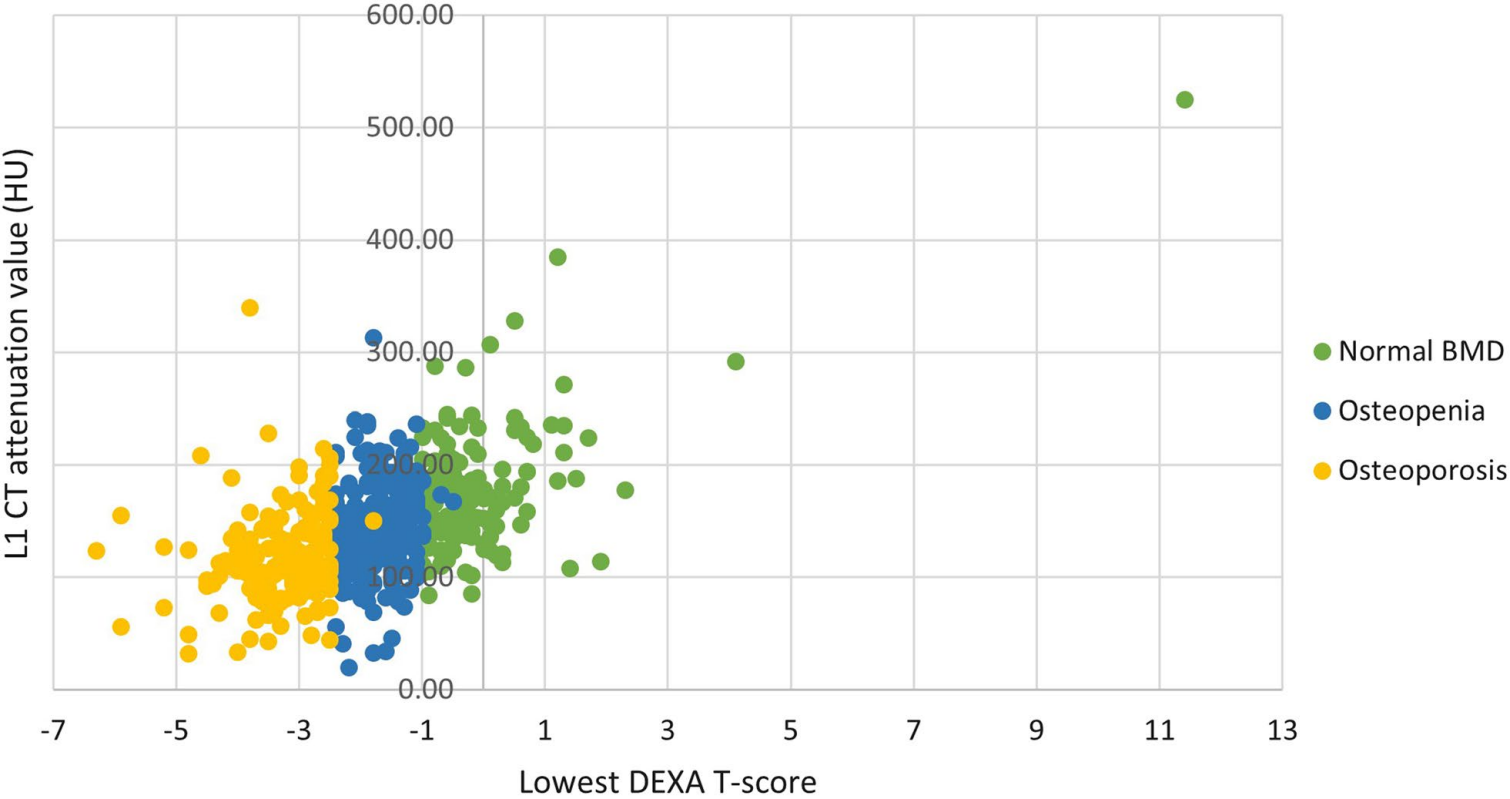
Scatterplot of L1 CT attenuation values (HU) and lowest recorded DEXA T-scores, separated according to DEXA-defined categories

There was correlation between the mean CT attenuation values and DEXA T-scores, with a Pearson coefficient of 0.55 (Fig. 3).

**Fig. 4:**
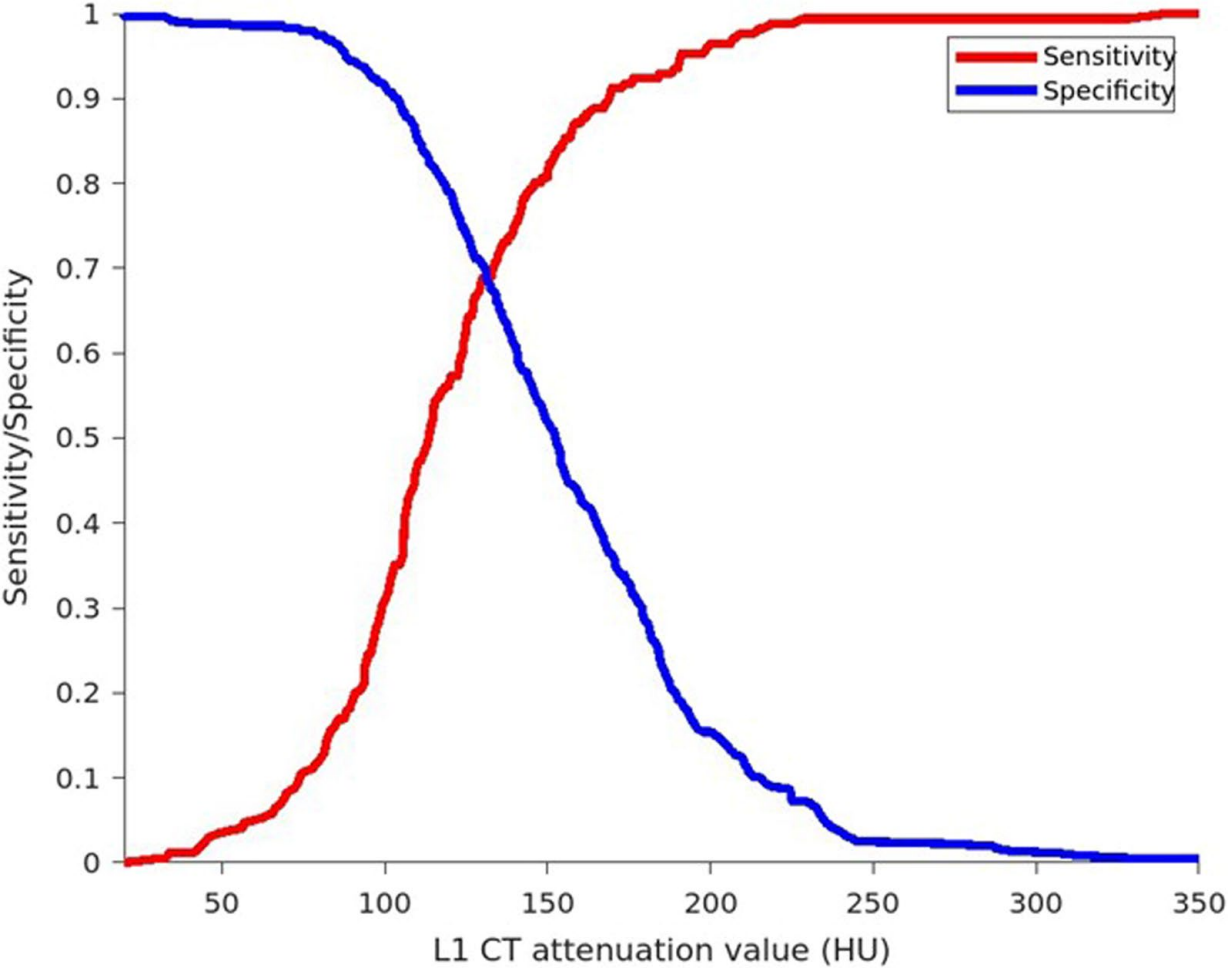
The graph shows the sensitivity and specificity trade-off at different CT attenuation thresholds. At higher thresholds, higher sensitivity is traded for lower specificity. A balanced sensitivity and specificity threshold was found at 131 HU

**Fig. 5:**
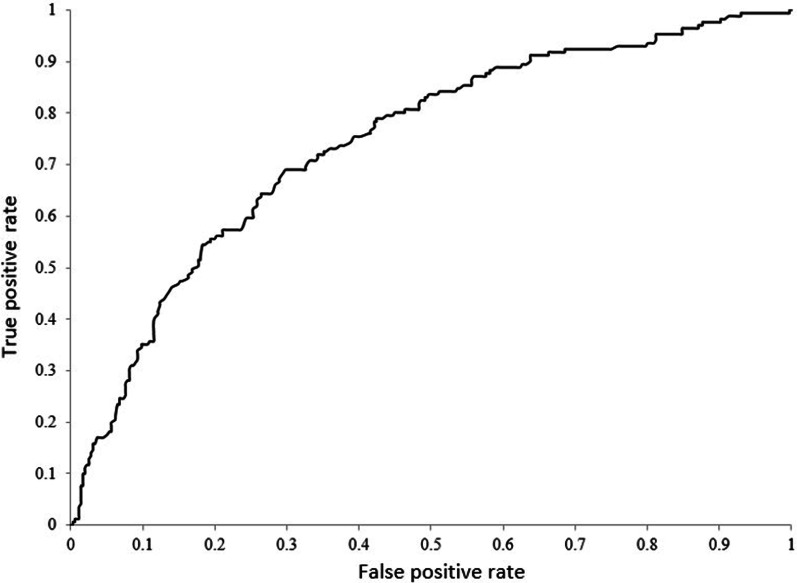
Receiver operating characteristic curve for predicting DEXA-defined osteoporosis using L1 CT attenuation values. The AUC is 0.74

We found that a threshold of 169 HU was 90% sensitive, and a threshold of 104 HU was 90% specific for distinguishing patients with osteoporosis from those with normal BMD or osteopenia. A threshold of 131 HU showed a balanced sensitivity and specificity of approximately 69% for each (Table 2, Fig. 4). We found an AUC of 0.74 (95% CI 0.69–0.78) (Fig. 5). At the threshold defined by Pickhardt as achieving 90% sensitivity (160 HU), we found a sensitivity of 88% and a specificity of 43%. At their threshold for achieving 90% specificity (110 HU), we identified a sensitivity of 48% and a specificity of 85%.

**Table 2:** Diagnostic performance of L1 CT attenuation values for distinguishing osteoporosis from osteopenia and normal BMD in the current study

	Threshold for achieving 90% sensitivity	Threshold for achieving 90% specificity	Threshold for balanced sensitivity and specificity
L1 CT attenuation, HU	≤ 169	≤ 104	≤ 131
Sensitivity, %	90	35	69
Specificity, %	36	90	70
PPV, %	41	63	52
NPV, %	89	75	83

*CT* computed tomography, *PPV* positive predictive value, *NPV* negative predictive value, *HU* Hounsfield units

## Discussion

Although previous studies have demonstrated the value of abdominal CT as a screening tool for osteoporosis, to our knowledge, this is the first study to assess this in a British population. The present study adds to the growing body of evidence that simple vertebral trabecular attenuation measures obtained from routine CT scans are a valuable tool in opportunistic screening for osteoporosis.

Lee et al. showed that CT attenuation measures obtained from the transverse and sagittal reconstructions were in agreement [23]. We chose to use the sagittal reconstruction for analysis of CT attenuation as the lumbar vertebrae are easily identified and common pathologies such as fractures can be easily visualised. Utility of a simple ROI measure has previously been shown to be as effective as phantomless quantitative CT measures, with good interobserver reproducibility [24].

It has previously been demonstrated that there is no significant difference in HU measures between the lumbar vertebrae [15, 25]. The L1 vertebra has been highlighted as a suitable landmark as measures taken from this location are as accurate, if not more so, than measures obtained at other vertebral levels. Furthermore, it is easily identifiable, thus improving reproducibility of measurements [14]. For this reason, we selected the L1 vertebra as an appropriate landmark where radiologists could easily perform a simple attenuation measure, with minimal effort and negligible time investment.

Previous studies have shown no significant difference in outcome whether the CT scan is taken pre- or post-contrast [14, 25, 26]. In view of this, we gathered data both pre- and post-contrast from different scanners, thus easily transferable to the real world.

Optimal cut-off values in other populations have previously been proposed. Pickhardt et al. suggested a threshold of ≤ 110 HU as 90% specific for distinguishing osteoporosis from non-osteoporosis, and a threshold of ≤ 160 HU as 90% sensitive. They did find a higher diagnostic performance, with an AUC of 0.83 and identified a balanced threshold of ≤ 135 HU as having around 75% sensitivity and specificity [14]. This contrasts with our study, which found a lower diagnostic performance, with an AUC of 0.74 and a balanced threshold of ≤ 131 HU as having 69% sensitivity and specificity. However, there has been previous evidence of discrepancies across studies [27]; for example, lower 90% specificity thresholds have previously been demonstrated, e.g. ≤ 73 HU [15]. This highlights likely variable performance of CT for osteoporosis screening across various settings and populations. This may be partly attributable to differences in scanning equipment and protocols [28]; nonetheless, our findings are in agreement with numerous other studies that have also shown good correlation between CT attenuation and BMD measured by DEXA, albeit with a lower diagnostic performance than reported by Pickhardt et al. [17, 20, 25, 27].

We propose that a higher threshold of ≤ 169 HU, which showed 90% sensitivity in our population, could be useful in high-risk cohorts to minimise false negatives. Meanwhile, a lower threshold of ≤ 104 HU may be of use in low-risk cohorts to minimise false positives and reduce the burden of unnecessary referrals. We recommend that radiologists liaise with primary care and rheumatology providers to determine appropriate cut-off thresholds for further investigation. Our research concurs with recommendations from a recent United Kingdom audit emphasising the importance of identifying patients with osteoporotic vertebral fragility fractures on CT studies [29].

The use of routine abdominal CT in opportunistic screening for osteoporosis presents various advantages over DEXA. One major advantage is the potential scalability of the method; it is possible to retrospectively assess numerous patients via the picture archiving and communication system, and BMD evaluation could also be applied on a routine basis in prospective cohorts. Once sufficient refinements to the technique have been made, it may be possible to incorporate the CT attenuation measures into risk assessment tools, such as the FRAX tool.

A disadvantage of DEXA is the under-utilisation of the technique, particularly in younger patients. Opportunistic use of abdominal CT enables screening to be applied to a much larger and more diverse population, thus enabling reduction of fracture risk on a much wider scale. Given the increasing frequency of CT imaging, this provides an ideal opportunity to deliver added value to patient care.

Another advantage of CT over DEXA is the rapid evaluation method requiring minimal effort from the radiologist. In the long run, we envisage that this could be a potential application for artificial intelligence, alerting radiologists that referral to rheumatology may be indicated in cases where BMD falls below a certain threshold. Automated algorithms have demonstrated favourable comparison to manual measurements of CT attenuation for evaluation of bone and muscle disease [30, 31]. However, comparisons of manual and automated measurements at the L1 vertebra showed a discrepancy of 21HU between the two methods, emphasising the need for rigorous validation of machine algorithms prior to incorporation in clinical practice [32].

We must also acknowledge the limitations of this study. One limitation is the inherent selection bias associated with including a population of patients who have undergone both DEXA and CT. As they are already undergoing investigation for osteoporosis, they naturally are likely to represent a population of interest. Another limitation is that we did not assess reproducibility; our reasoning is that previous studies have shown reliable interobserver measurements [23]. We acknowledge that DEXA T-scores are often obtained from the hip, whilst our study is based on vertebral CT attenuation measures and also does not consider other factors utilised in fracture risk assessment scores.

The number of patients with vertebral fractures was not assessed in this study, as we felt that patients with a vertebral fracture should be referred for full osteoporosis assessment in a similar manner to those with a L1 attenuation value below an agreed threshold. Previous studies have shown that DEXA scans frequently produce false negative results in patients with osteoporosis-related vertebral collapse fractures [14, 15].

## Conclusion

In conclusion, we suggest there is scope for L1 vertebral attenuation measures to be incorporated as a rapid and effective tool in the United Kingdom to opportunistically screen for osteoporosis. This research provides a framework for future controlled studies in the United Kingdom that, in collaboration with rheumatologists and primary care providers, could be used to determine suitable threshold values that may be incorporated into routine clinical practice: thresholds would likely depend on local factors such as capacity and resource availability. Earlier diagnosis of osteoporosis would enable timely commencement of treatment and thus prevention of fragility fractures, with a long-term effect of improving morbidity and mortality. This work aligns with the recommendations of the recent United Kingdom national audit on osteoporotic vertebral fragility fractures on CT [29].

## Data Availability

The datasets used and/or analysed during the current study are available from the corresponding author on reasonable request.

## Abbreviations

BMD: Bone mineral density
CT: Computed tomography
CI: Confidence interval
DEXA: Dual-energy X-ray absorptiometry
HU: Hounsfield units
NPV: Negative predictive value
PPV: Positive predictive value
ROC: Receiver operating characteristic
ROI: Region of interest
SD: Standard deviation
